# Knockdown of C3aR alleviates age-related bone loss *via* activation of YAP1/β-catenin signaling

**DOI:** 10.1016/j.jbc.2025.108500

**Published:** 2025-04-09

**Authors:** Fangyu Li, Shun Cui

**Affiliations:** 1 Department of Rheumatology and Immunology, Union Hospital, Tongji Medical College, Huazhong University of Science and Technology, Wuhan, China; 2 Department of Endocrinology, Union Hospital, Tongji Medical College, Huazhong University of Science and Technology, Wuhan, China

**Keywords:** bone homeostasis, C3aR, age-related bone loss, JR14a, Yap/β-catenin

## Abstract

The complement system plays an important role in bone growth during physiological development and skeletal homeostasis. However, the specific impact of the complement C3a receptor (C3aR) on age-related bone loss remains unclear. In this study, we found that C3aR expression increased with age and was the same as that of the senescent molecules p53, p21, and p16 in control mice. Knockdown of C3aR reduced the expression of senescence markers and significantly ameliorated bone senescence. Notably, C3aR knockdown in mice effectively reversed age-induced bone loss, which was characterized by an increase in the number of osteoblasts and a decrease in the number of osteoclasts. In an *in vitro* model of D-gal-induced senescence, increased expression of C3aR correlated with increased expression of senescence markers such as p53, p21, and p16. Treatment with a C3aR antagonist (JR14a) successfully attenuated the expression of these markers of cellular senescence and reduced the proportion of late apoptotic cells. Mechanistically, JR14a treatment mitigated D-gal-mediated inhibition of osteoblastic differentiation in preosteoblasts through activation of the YAP1/**β**-catenin signaling pathway. In the D-gal-induced aging mouse model, treatment with JR14a ameliorates bone microarchitecture and bone loss. In summary, these studies revealed a role for C3aR in regulating bone homeostasis, suggesting that targeting C3aR may be a promising therapeutic strategy for the treatment of age-related osteoporosis.

Osteoporosis is a common disease in elderly individuals, and its prevalence has increased globally with the ageing of the population (1, 2). Bone is a dynamic tissue that maintains a balance of bone remodeling through the interregulation of bone formation by osteoblasts and bone resorption by osteoclasts, which is essential for the efficient and lifelong performance of vital skeletal functions (3, 4). With ageing, bone remodeling becomes dysfunctional, with decreasing osteoblast activity and prolonged osteoclast lifespan, favoring bone resorption and resulting in bone loss, which ultimately leads to osteoporosis and fragility fractures (5, 6, 7). There is growing awareness that ageing itself accelerates bone loss *via* mechanisms such as cellular senescence and oxidative stress (8). Therefore, delaying ageing, reducing bone resorption, and promoting bone formation are essential for the treatment of age-related osteoporosis.

The complement system is a major component of the immune system, providing the first-line defense against infection (9). Complement proteins are believed to be critical not only in activated immune system states but also for bone growth during physiological development and bone homeostasis (10). The complement system has been implicated in the bone remodeling-related shift from bone formation to resorption and subsequent bone loss in numerous studies (11, 12, 13). C3aR is an ∼ 100 kDa G protein-coupled receptor that is expressed in bone marrow cells, including bone marrow mesenchymal stem cells (BMSCs), OBs, monocytes/macrophages, and OCs, indicating a potential role for C3aR in the regulation of bone homeostasis (14, 15, 16). Recently, it was reported that C3a/C3aR stimulates the secretion of proinflammatory cytokines, such as TNF-α and MMP9, through the transcription factors NF-kB and NFATc1, leading to osteoclast differentiation and proliferation *in vivo* and *in vitro* (17). Thus, C3aR plays an important role in bone biology, regulating the differentiation of OCs. However, the effect of C3aR on age-related bone loss has not been reported. Therefore, further studies on the effects of C3aR on bone homeostasis are needed.

In this study, we detected increased expression of C3aR in the femurs of aged mice and identified C3aR as an important target involved in age-related bone loss. Aged C3aR^ko^ mice presented increased bone formation, decreased bone marrow adipocytes, decreased bone resorption, and increased bone mass. In addition, the *in vitro* addition of a highly selective C3a receptor antagonist(JR14a) prevented D-gal-induced senescence of preosteoblasts(MC3T3-E1) and promoted osteoblast differentiation. Collectively, these results suggest that C3aR is involved in the regulation of bone homeostasis and skeletal senescence and is a potential target for the treatment of age-related bone loss.

## Results

### C3aR expression is upregulated in bone with aging

**Figure 1:**
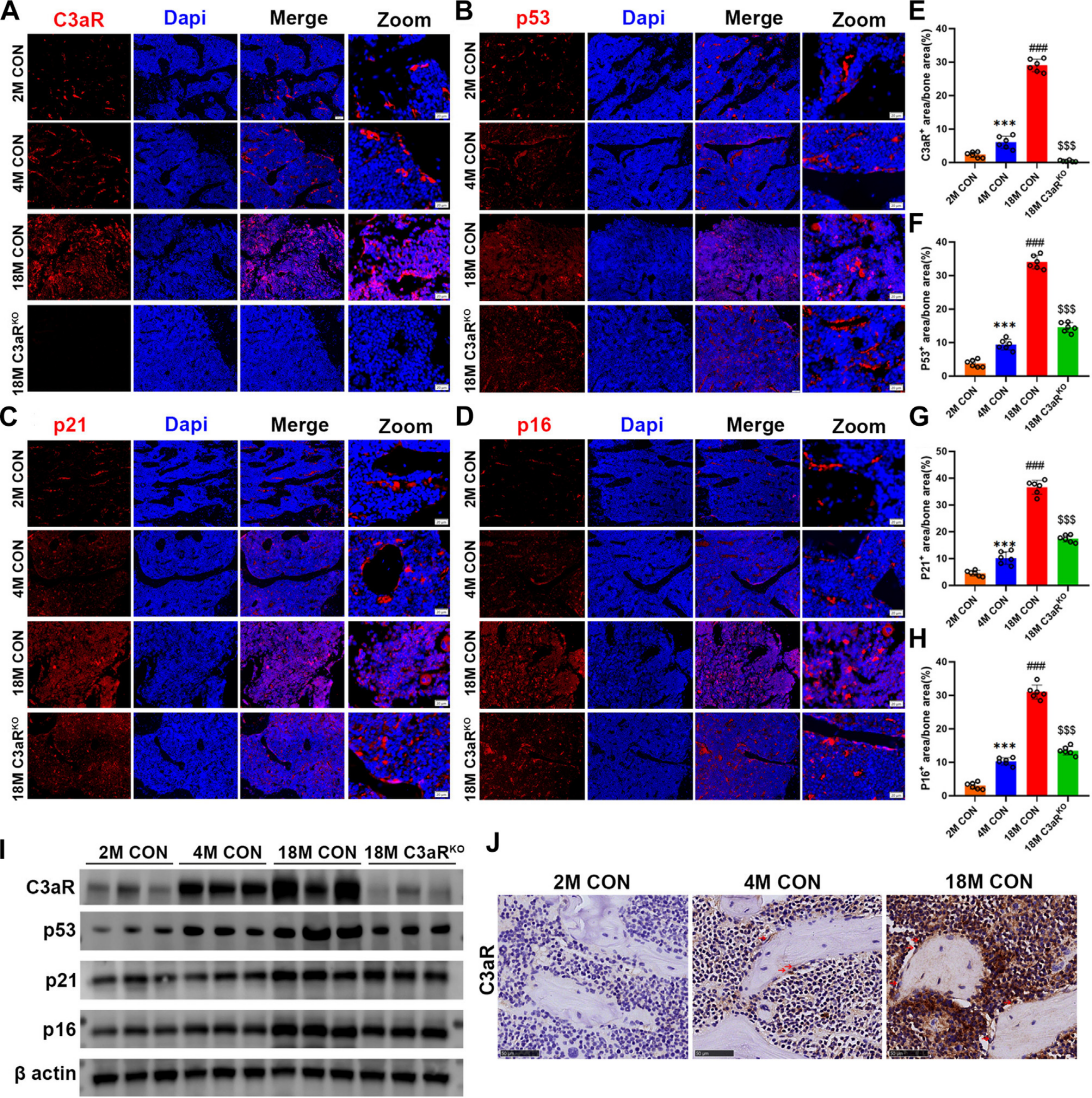
**C3aR expression is upregulated in skeletal bones with aging.***A, B, E*, and *F*, representative immunofluoresce images of C3aR, p53, p21, and p16 in the bones of 2-, 4-, and 18-month-old littermate control mice and 18M C3aR^ko^ group mice. Scale bars = 50 μm. *C, D, G*, and *H*, quantitative analysis of C3aR-, p53-, p21-and p16-positive osteocytes. In zoom images, Scale bars = 20 μm. *I*, protein expression of C3aR, p53, p21, and p16 in mouse bones. *J*, representative immunohistochemical images of C3aR in the bones of 2-, 4-, and 18-month-old littermate control mice. The values are expressed as the means ± standard deviations (n = 5 or n = 6). ∗∗∗*p* < 0.001 compared with 2 M CON mice. ^###^*p* < 0.001 compared with 4 M CON mice.^$$$^*p* < 0.001 compared with 18 M CON mice.

To investigate the relationship between C3aR and age-related bone loss, we examined the expression of C3aR and senescence markers (p16, p21, p53) in the skeletal bones of 2-, 4- and 18-month-old mice. C3aR expression was significantly greater in the bones of 18-month-old aged male mice than in those of young mice and exhibited the same expression pattern as the ageing-related markers p16, p21, and p53 (Fig. 1, *A*–*H*). Because bone tissue is a complex system composed of different types of cells, including osteoblasts, osteoclasts and osteoclasts as well as hematopoietic cells and other various supporting cells (18). We further examined the location of C3aR and senescence marker expression in bone tissue. In 2- and 4-month-old control mice, C3aR and senescence markers were expressed on osteoblasts (rectangular shape) and osteoclasts (multinucleated fusion) (Fig. 1, *A*–*D*). In 18-month-old control mice, C3aR and senescence markers were expressed on multiple cell types (Fig. 1, *A*–*D*).

We hypothesized that Knockdown of C3aR attenuates cellular senescence in bone. Western blot analysis revealed that the expression of p16, p21, and p53 was also lower in the femurs of aged C3aR^ko^ mice than in those of aged mice from the same litter (Fig. 1*I*). Consistent with the protein levels, immunofluorescence revealed decreased positive expression of p16, p21, and p53 in the bones of aged C3aR^ko^ mice (Fig. 1, *B*–*H*). Next, *via* immunohistochemistry (IHC) staining, we observed increased expression of C3aR with age, especially in osteoblasts (Fig. 1*J*, red arrows).

These results indicate that the expression of C3aR and senescence-associated proteins (p16, p21, and p53) increases with age. Knockdown of C3aR ameliorated the age-associated increased expression of p16, p21, and p53 in the femur. In addition, C3aR is expressed on osteoblasts, osteoclasts, and other stromal cells. Here, we focused on representative bone remodeling processes of osteoclast resorption and osteoblast formation.

### Knockdown of C3aR increases bone mass in aged mice

**Figure 2:**
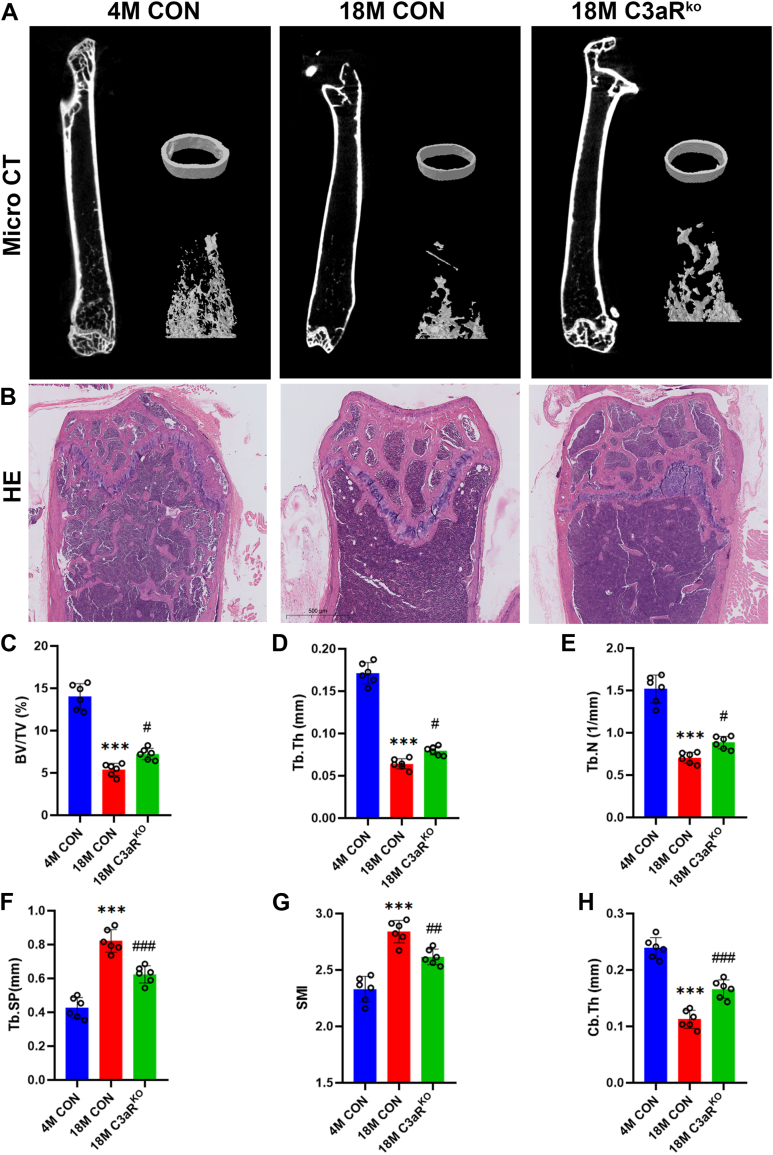
**Knockdown of C3aR increases bone mass in aged mice.***A*, representative images of μCT reconstructions of femoral trabecular and cortical bones from 4- and 18-month-old littermate control mice and 18 M C3aR^ko^ group mice. *B*, HE staining of femurs from the three groups. Scale bars = 500 μm. *C, D, E, F, G, and H*, quantification of the trabecular bone volume per total volume (BV/TV), trabecular number (Tb.N), trabecular separation (Tb.Sp), trabecular bone thickness (Tb.Th), structural model index (SMI) and cortical bone thickness (Cb.Th). ∗∗∗*p* < 0.001 compared with 4 M CON mice; ^#^*p* < 0.05, ^##^*p* < 0.005, ^###^*p* < 0.001 compared with 18 M CON mice.

Next, we performed three-dimensional reconstruction of the distal femur *via* microcomputed tomography (μCT), followed by epiphyseal reconstruction and trabecular morphometric parameter analysis to investigate the differences in femoral bone mass between C3aR^ko^ mice and littermate control mice. Compared with the 4 M control group, the 18 M control group presented a significant reduction in bone mass, destruction of the bone microarchitecture, and disorganization of the trabecular bone structure. μCT analysis revealed increased bone mass in aged C3aR^ko^ mice compared with 18 M control mice, as evidenced by increased BV/TV, Tb.N, Tb.Th, and Cb.Th as well as a decrease in Tb.Sp (Fig. 2, C–*H*). HE staining revealed that aged C3aR^ko^ mice had more bone trabeculae than age-matched littermate control mice did (Fig. 2*B*).

### Increased bone formation and attenuated bone resorption in C3aR knockout mice

**Figure 3:**
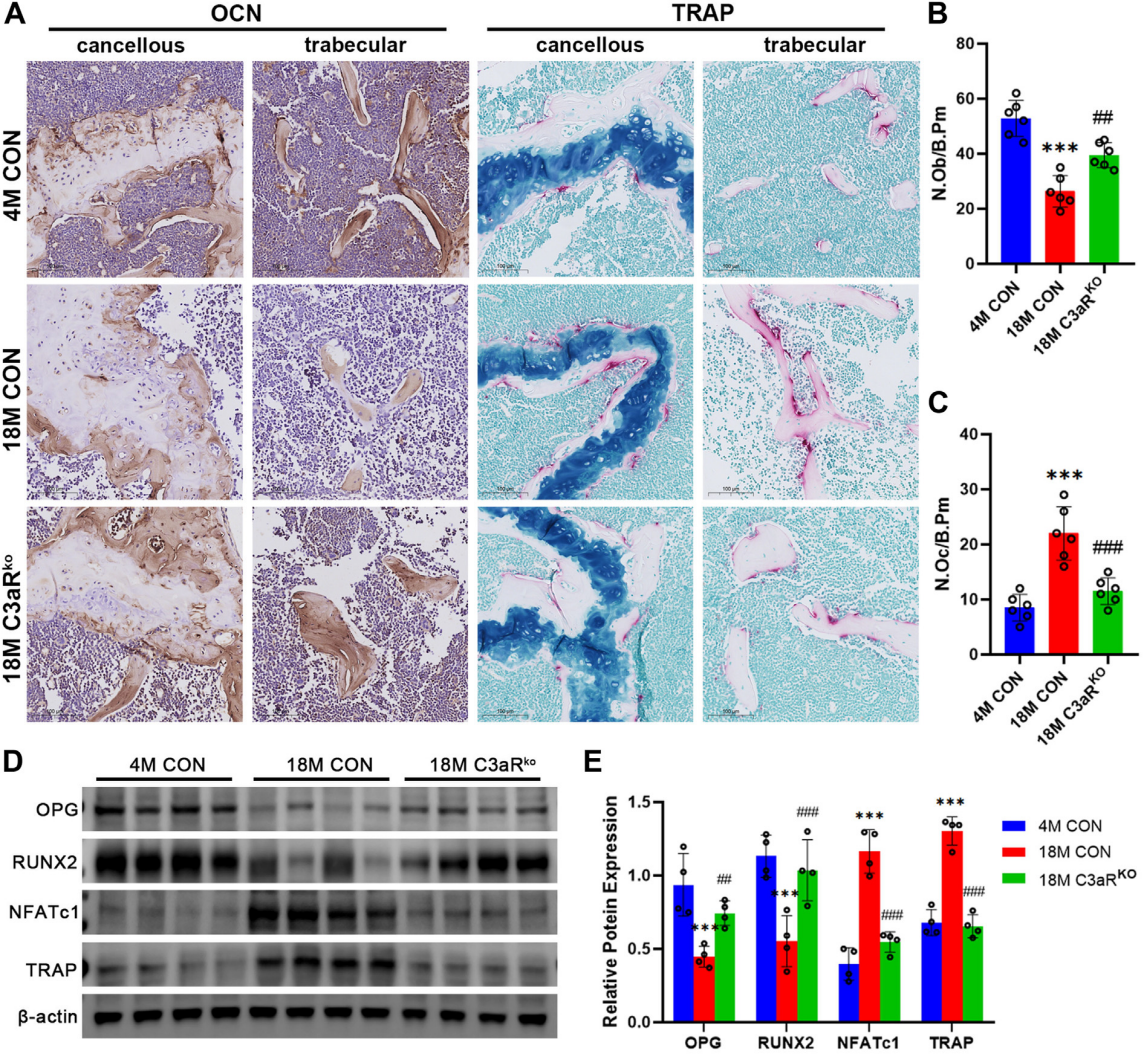
**Increased bone formation and attenuated bone resorption in C3aR knockout mice**. *A*, representative image of OCN and TRAP staining in bone tissue (cancellous and trabecular). bars = 100 μm. *B* and *C*, the number of OCN-positive osteoblasts per bone perimeter (N.Ob/B.Pm). The number of osteoclastic cells per bone perimeter (N.Oc/B.Pm). *D* and *E*, protein expression of OPG, RUNX2, NFATc1, and TRAP in mouse bones and densitometric quantification. The values are expressed as the means ± standard deviations (n = 5 or n = 6). ∗∗∗*p* < 0.001 compared with 4 M CON mice; ^#^*p* < 0.05, ^##^*p* < 0.005, ^###^*p* < 0.001 compared with 18 M CON mice.

Therefore, we examined the expression of osteocalcin (OCN) and tartaric acid resistance phosphatase (TRAP) in the femurs of C3aR^ko^ mice and 4 M,18 M littermate control mice. IHC staining revealed that, compared with 4 M control mice, 18 M mice presented decreased skeletal OCN expression and increased positive TRAP expression. Moreover, femoral OCN expression was increased, and TRAP expression was decreased in 18MC3aRko mice compared with 18 M control mice (Fig. 3, *A*–*C*). Similarly, with ageing, the expression of osteogenic marker proteins (osteoprotegerin (OPG) and runt-related transcription factor 2 (RUNX2)) decreased, and the expression of marker molecules for osteoclasts (nuclear factor of activated T cells cytoplasmic 1 (NFATc1) and TRAP) increased in the bones of 18 M mice (Fig. 3, *D* and *E*). In contrast, 18 M C3aR^ko^ mice presented increased expression of osteogenic markers and decreased expression of osteoclast marker molecules compared with 18 M controls (Fig. 3, *D* and *E*).

### A C3aR antagonist ameliorates D-gal-induced senescence in preosteoblasts

**Figure 4:**
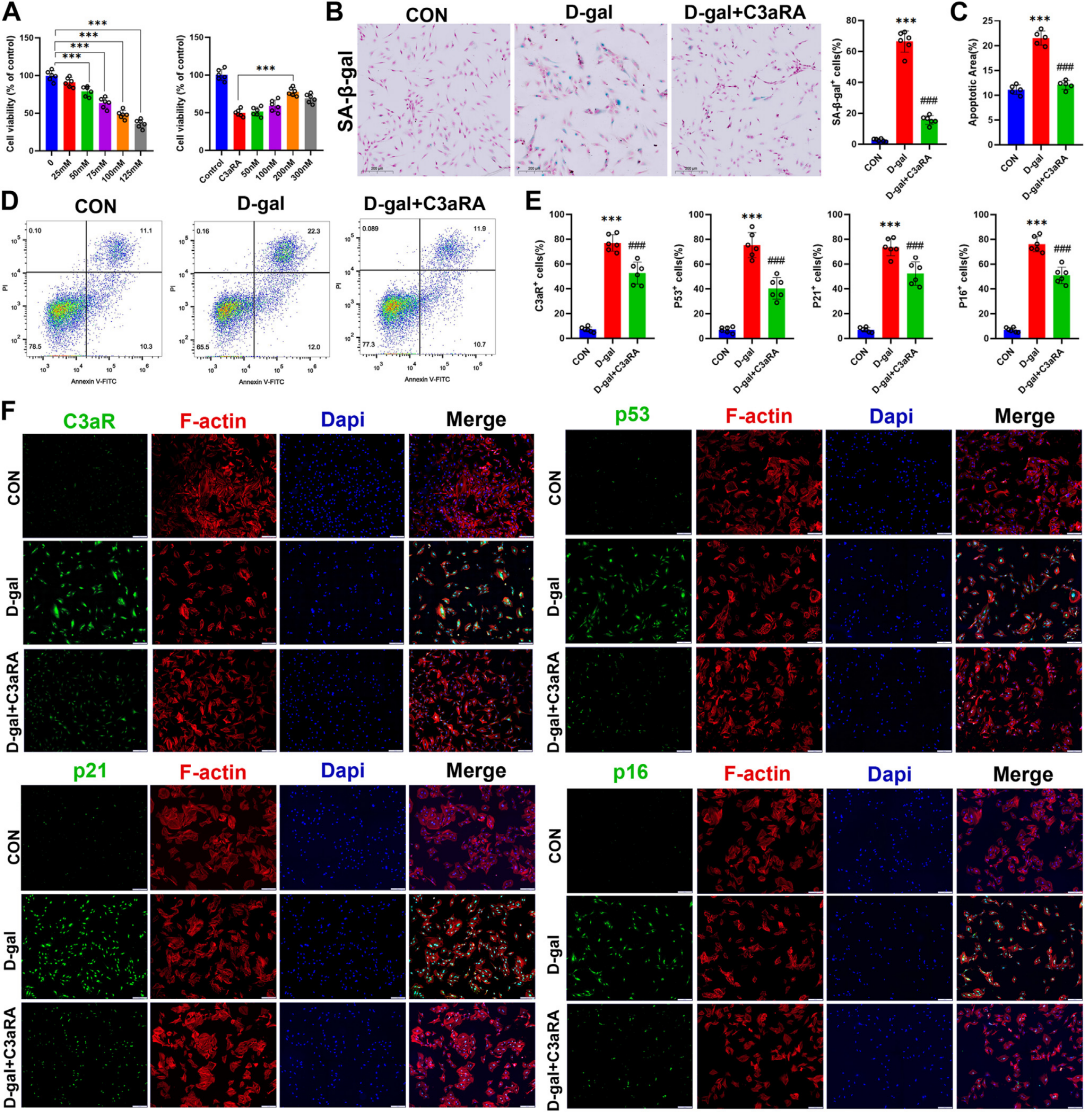
**A C3aR antagonist ameliorates D-gal-induced senescence in preosteoblasts.***A*, viability assessment of MC3T3-E1 cells under varying concentrations of D-gal and C3aR antagonists using CCK-8. *B*, SA-β-gal staining and quantitative analysis. MC3T3-E1 cells were cultured in media supplemented with D-gal (100 mM) or D-gal + C3aRA (JR14a, a novel antagonist of C3aR, 200 nM) for 48 h. Scale bar = 200 μm. *C, D*, flow cytometry analysis of apoptosis in the different groups. *E, F*, representative immunofluorescence images of C3aR, p53, p21, and p16 expression and quantitative analysis. ∗∗∗*p* < 0.001 compared with the CON group. ^###^*p* < 0.001 compared with the D-gal group.

To assess the role of C3aR in senescent preosteoblasts, we constructed a model of senescence *via* D-gal stimulation of MC3T3-E1 cells and treated them with a C3aR antagonist (JR14a). First, cell viability was assessed using CCK8 after 48 h of intervention with different concentrations of D-gal and C3aR antagonist. The results showed that when the concentration of D-gal was 100 mM, the cell viability decreased to about 50%, indicating that this concentration had a significant inhibitory effect on the cells and could be used as a suitable intervention concentration for subsequent experiments. In addition, after further addition of 200 nM C3aRA on top of 100 mM D-gal intervention, the cell viability was significantly restored to a relatively desirable level, indicating that C3aRA has a protective effect against D-gal-induced cell damage at this concentration (Fig. 4*A*). SA-β-Gal staining revealed that D-gal exposure induced senescence in MC3T3-E1 cells, and the use of a C3aR antagonist (C3aRA group) reduced cellular senescence compared with that in the D-gal group (Fig. 4*B*). Flow cytometry results revealed that C3aR antagonist treatment decreased the proportion of D-gal-induced late apoptotic cells (Fig. 4, *C* and *D*). Similarly, the immunofluorescence results revealed that the C3aR antagonist reduced D-gal-induced expression of the cellular senescence markers p16, p21, and p53 (Fig. 4, *E* and *F*).

### JR14a improves osteoblast differentiation through activation of YAP1/**β**-catenin signaling

**Figure 5:**
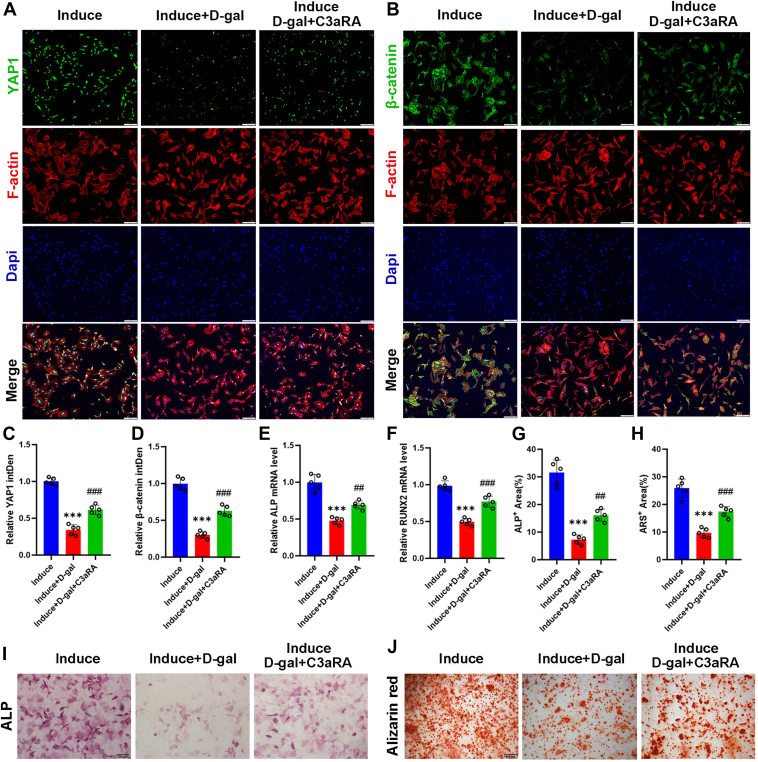
**JR14a improves osteoblast differentiation through activation of YAP1/β-catenin signaling.***A*, Representative fluorescence images of F-actin (*red*) and yap (*green*) double staining in MC3T3-E1 cells. Scale bar = 200 μm. *B*, representative fluorescence images of F-actin (*red*) and β-catenin (*green*) double staining in MC3T3-E1 cells. Scale bar = 200 μm. *C, D*, quantitative analysis. (E, F) Relative mRNA levels. *G, H*, quantitative analysis of ALP staining and Alizarin *red* staining. *I*, ALP staining after osteoblast differentiation for 7 days. Scale bar = 200 μm. *J*, Alizarin *red* staining after osteoblast differentiation for 14 days. Scale bar = 500 μm. ∗∗∗*p* < 0.001 compared with the Induce group. ##*p* < 0.01, ###*p* < 0.001 compared with the Induce D-gal group.

The YAP1/β-catenin pathway is of great interest in osteoblasts and is activated by the addition of osteoblast differentiation-inducing fluids. The addition of D-gal to the induction medium significantly inhibited the activation of YAP1 and β-catenin, which was reversed by the use of a C3aR antagonist (Fig. 5, *A*–*D*). After 3 days of culture, the expression of the osteogenic differentiation marker genes *ALP* and *RUNX2* was significantly lower in the Induce + D-gal group than in the Induce group, which was partially reversed by treatment with the C3aR antagonist (Fig. 5, *E* and *F*). ALP staining and alizarin red staining were performed on days 7 and 14 of osteogenic induction, respectively. The staining results showed that the Induce D-gal group resulted in a decreased osteogenic differentiation capacity and a reduced area of positive ALP and alizarin red staining, compared with the Induce group (Fig. 5, *G*–*J*). C3aR antagonist ameliorated the decreased osteogenic capacity caused by D-gal.

### JR14a treatment attenuates D-gal-induced bone loss

**Figure 6:**
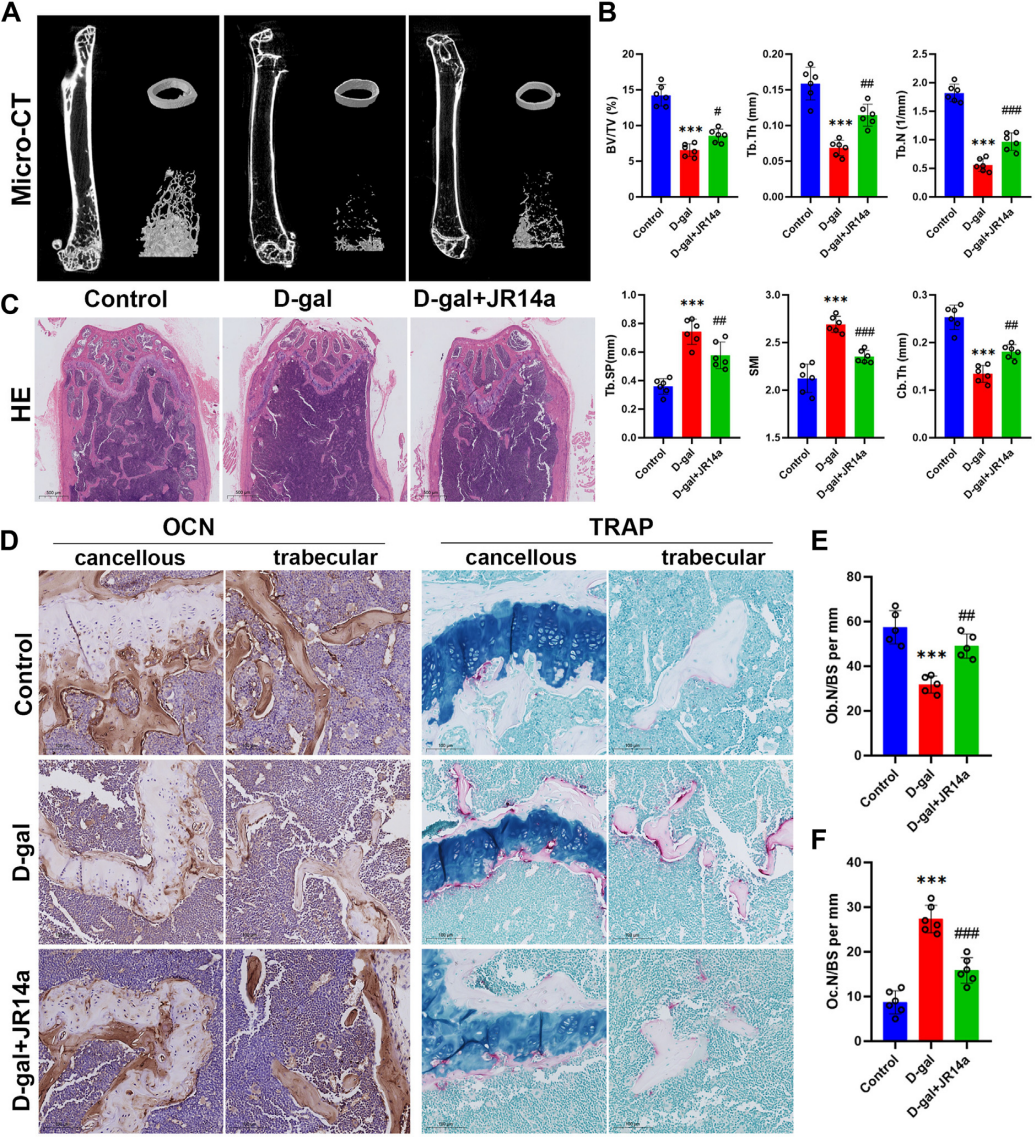
**JR14a treatment attenuates D-gal-induced bone loss.***A*, representative images of μCT reconstructions of femoral trabecular and cortical bones from control, D-gal, and D-gal + JR14a group mice. *B*, quantification of the trabecular bone volume per total volume (BV/TV), trabecular number (Tb.N), trabecular separation (Tb.Sp), trabecular bone thickness (Tb.Th), structural model index (SMI) and cortical bone thickness (Cb.Th). *C*, HE staining of femurs from the three groups. Scale bars=500 μm. *D*, representative image of OCN and TRAP staining in bone tissue (cancellous and trabecular). bars = 100 μm. *E, F*, the number of OCN-positive osteoblasts per bone perimeter (N.Ob/B.Pm). The number of osteoclastic cells per bone perimeter (N.Oc/B.Pm). ∗∗∗*p* < 0.001 compared with Control group; ##*p* < 0.005, ###*p* < 0.001 compared with D-gal group.

To further elucidate the role of JR14a in D-gal-induced bone loss, 8-week-old wild-type mice received DMSO or D-gal intervention for 8 weeks, where the D-gal + JR14a group received 4 weeks of JR14a treatment. Micro-CT analysis showed bone microstructural disruption in the D-gal group, as evidenced by a decrease in BV/TV, Tb.Th,Tb.N, and Cb.Th, and an increase in Tb.SP and SMI. These findings were further confirmed by histologic staining, which showed decreased bone trabecular area, disordered arrangement, decreased osteoblast numbers, and increased osteoclast numbers in the D-gal group compared with the Control group (Fig. 6). Treatment with JR14a partially reverses D-gal-induced bone loss by increasing the number of osteoblasts, decreasing the number of osteoclasts, and improving bone microarchitecture (Fig. 6).

## Discussion

The complement system plays an important role in bone growth and bone homeostasis during physiological development; however, the role of C3aR in age-related bone loss is unknown. In this study, we found that C3aR expression increased in bone with age and that the knockdown of C3aR ameliorated skeletal ageing in mice. Compared with age-matched littermate control mice, aged C3aR^ko^ mice presented increased bone formation, decreased bone resorption, and increased bone mass. Moreover, the *in vitro* addition of a highly selective C3a receptor antagonist (JR14a) prevented D-gal-induced senescence of preosteoblasts (MC3T3-E1) and activated YAP1/β-catenin signaling to promote osteoblast differentiation. Treatment with JR14a ameliorates D-gal-induced bone microarchitectural disruption and bone loss. These results suggest that C3aR is involved in the regulation of bone homeostasis and skeletal senescence and is a potential target for the treatment of age-related osteoporosis.

Changes in the complement system have long been recognized to occur with normal aging and inflammation plays a central role in a variety of ageing-related diseases (19). Multiple studies have shown the that levels of C3 and C3aR are elevated in the brain in ageing and Alzheimer's disease (AD) mouse models, as well as in patients with AD, and that their inactivation prevents age-related functional decline and AD neuropathology (20, 21, 22). Age-related bone loss is highly correlated with senescence. The senescence-related genes p16 and p21 play important roles in the initiation of senescence by causing cell cycle arrest (23, 24). Interestingly, our findings revealed an increase in skeletal C3aR expression with increasing age, with a similar expression pattern to that of the ageing markers p53, p21, and p16. Moreover, C3aR knockdown significantly improved these senescence characteristics. In addition, our study provides insight into the relationship between C3aR and osteocyte senescence. In an *in vitro* model in which D-gal was uesed to induce senescence in preosteoblasts, the increase in C3aR expression coincided with an increase in the levels of senescence markers such as p53, p21, and p16. Treatment with a specific C3aR antagonist (JR14a) effectively attenuated the expression of these markers, suggesting that C3aR signaling plays a crucial role in regulating the osteoblast senescence pathway. This mechanistic study not only links complement activation to the cellular senescence process but also identifies potential therapeutic targets for alleviating age-related bone degeneration.

The formation of new bone by osteoblasts and the resorption of old bone by osteoclasts are key processes in the maintenance of bone homeostasis and are compromised with age and pathological conditions such as fracture (6). In a fracture healing study, localized C3 expression was increased in damaged bones, and C3/C3a further induced proinflammatory cytokines such as TNF-α and MMP9, leading to osteoclast differentiation and proliferation *in vitro*, which in turn delayed fracture healing (17). C3 is required for the proliferation of precursor cells and early differentiation of osteoclasts and promotes osteoclast formation (13, 14). In contrast, blocking C3 with an anti-C3 antibody significantly inhibited osteoclastogenesis *in vitro* (25). Moreover, the addition of a C3aR antagonist *in vitro* effectively reduced osteoclast cluster formation (17). In the complement system, C3 and its cleavage product C3a have emerged as pivotal regulators of bone metabolism. MacKay *et al.* demonstrated that C3 plays an important role in the model of ovariectomy-induced osteoporosis. Compared with wild-type mice, C3-deficient mice showed significantly reduced bone loss and improved bone mechanical properties after ovariectomy (13). Similarly, Kuhn *et al.* found that C3aR^−/−^ mice had increased bone mass, increased osteoblast activity, and decreased osteoclastic cells compared to wild-type mice (26). These findings are consistent with our study, which showed increased bone formation, decreased bone resorption, and increased bone mass in C3aR^ko^ mice compared with littermate controls. Furthermore, treatment with the C3aR antagonist JR14a effectively mitigated D-galactose-induced bone microarchitectural disruption and bone loss. Interestingly, Matsuoka *et al.* reached a differing conclusion that daily administration of SB290157 to block the effects of C3a exacerbated bone loss during the high bone conversion state of ovariectomy (OVX) (27). This discrepancy in the effects of C3aR antagonists on bone loss may be closely related to the distinct bone metabolic states involved. OVX-induced osteoporosis is characterized by a high bone turnover state, where both osteoblast and osteoclast activities are significantly elevated (28). In this state, C3aR antagonists like SB290157 may reduce the stimulation of osteoblastogenesis by disrupting osteoblast-osteoclast coupling, ultimately leading to increased bone loss. Conversely, age-related bone loss represents a low bone turnover state, marked by decreased activity of both osteoblasts and osteoclasts (29). In this context, C3aR antagonists may exert their effects through different mechanisms, potentially by modulating cellular senescence or other age-related pathways. In addition, this study revealed that a C3aR antagonist activated YAP1/β-catenin signaling, which was inhibited by D-gal during osteogenesis. These findings imply that pharmacological inhibition of C3aR could prevent or alleviate age-related bone loss by promoting osteoblast function and inhibiting osteoclast activity, thereby preserving bone density and strength in ageing individuals. Future studies may further elucidate the clinical implications of C3aR modulation in treating skeletal disorders associated with ageing and senescence.

### Conclusions

Our results suggest that C3aR plays an important role in age-related bone loss. C3aR antagonists promote osteoblastogenesis by ameliorating preosteoblast senescence and activating YAP1/β-catenin signaling.

## Experimental procedures

### Animals

Homozygous C3aR knockout mice (B6.129S4(C)-C3ar1^tm1Cge^/J, #033904; C3aR^ko^) on the C57BL/6J background were purchased from The Jackson Laboratory. These mice were genetically characterized *via* genomic DNA isolated from tail biopsies. C57BL/6J mice were purchased from the Model Animal Research Center of Nanjing University. C3aR^ko^ mice were backcrossed to the C57BL/6 background for at least eight generations, and littermate mice were used as the control group. All animal experimental procedures were reviewed and approved by the Institutional Animal Care and Use Committee at Tongji Medical College, Huazhong University of Science and Technology (IACUC number: 2538). The mice were housed in an environmentally controlled barrier animal facility (SPF level) with a 12-h light/12-h dark cycle and were fed ad libitum with mouse chow and water.

Six-week-old C57 mice were obtained from the Model Animal Research Center of Nanjing University and randomly divided into three groups (n = 6–8): control group, D-gal group (D-gal, 150 mg/kg/d); D-gal + JR14a group (D-gal, 150 mg/kg/d, JR14a, 2 mg/kg/d). The control group was continuously injected with saline containing DMSO for 8 weeks. D-gal was continuously injected for 8 weeks, and JR14a was continuously injected for the last 4 weeks.

### Micro-CT analysis

The three-dimensional structure of the trabecular bone was analyzed in femora *via* a micro-CT scanner (Skyscan-1176, Bruker, Karlsruhe, Germany). The system was configured with a 1 mm AL filter, 9 μm resolution, 50 kV voltage, and 200 μA current. For the femur, scanning started at the lower growth plate and extended proximally for 600 slices, which included trabecular bone (slices 1–400) and cortical bone (slices 500–550). The following parameters were measured with a CT Analyzer (version 1.15.4.0): bone volume (BV), total volume (TV), the trabecular bone volume fraction (BV/TV), the trabecular bone thickness (Tb.Th), the trabecular bone number (Tb.N), the trabecular bone separation (Tb.Sp), the structural model index (SMI), and the cortical bone thickness (Cb.Th).

### Immunohistochemistry and immunofluorescence

Bone histomorphometric analyses were performed as previously described (30, 31). In brief, mouse tibias and femurs were fixed overnight in 10% buffered formalin and decalcified in 14% EDTA for 21 days. After decalcification, femoral tissues were dehydrated in an ethanol gradient, cleared in xylene, embedded in paraffin, sectioned with a Leica slicer at a thickness of 4 μm, placed gently on the surface of warm water at 40 °C, dried at 60 °C, and stored at 4 °C until use.

For IHC and immunofluorescence (IF), the sections were incubated at 4 °C overnight with primary antibodies against C3aR(1:100, NBP2-15649, Novus), p53 (1:100, 60283-2-Ig, Proteintech), p21 (1:100, 10355-1-AP, Proteintech), p16 (1:100, 10883-1-AP, Proteintech), OCN (1:100, 23418-1-AP, Proteintech), YAP (1:100, 13584-1-AP, Proteintech), and β-catenin (1:100, 51067-2-AP, Proteintech). After being rinsed three times with PBS for 10 min each, the sections were incubated with the corresponding secondary antibodies for 1 h. For IF, the nuclei were stained with DAPI (Servicebio, Wuhan, China) for 10 min in the dark at room temperature. The sections were observed with a fully automated slice scanning system (VS120, Olympus, Japan).

TRAP staining (Wako, Tokyo, Japan) was performed according to the manufacturer's instructions.

### Cell culture and treatment

MC3T3-E1 cells were purchased from the National Collection of Authenticated Cell Cultures. The cells were cultured in α-minimal essential Eagle's medium (αMEM, Gibco) supplemented with 10% foetal bovine serum (FBS, Gibco) and 1% penicillin/streptomycin at 37 °C in a humidified atmosphere of 5% CO_2_. MC3T3-E1 cells were divided into several groups: the blank control group, D-gal group (D-gal, 100 mM (18 g/L), HY-N0210, MedChemExpress), and D-gal + JR14a (a novel antagonist of C3aR, 200 nM (106.7 μg/L), HY-138161, MedChemExpress) groups. After treatment for 48 h, the cells were collected for further analysis.

When the density of MC3T3-E1 in the 6-well plate reached 70%, the culture medium was replaced with osteogenic induction medium (MUXMX-90021, OriCell), and the medium was changed every 3 days. At day 7, staining was performed using the ALP staining kit (Sigma). At day 14, alizarin red staining was performed (OriCell).

### SA-**β**-gal staining

SA-β-gal staining was performed according to the manufacturer's instructions (#RG0039, Beyotime). X-ga1 (5-bromo-4-chloro-3-indolyl-β-D-galactoside) was used as a substrate in the staining, and the hydrolysis of the galactoside bond was catalyzed by senescence-specific β-galactosidase to produce a dark blue product. Images were taken with a bright field, and the percentage of SA-β-gal positive cells was expressed as the ratio of blue-stained cells to total cells.

### Western blotting

Total protein was extracted from mouse femurs *via* RIPA buffer containing protease and phosphatase inhibitors. After eliminating cell debris, the cell lysates were boiled for 5 min in SDS loading buffer; the proteins were then resolved *via* SDS–PAGE and transferred to polyvinylidene difluoride (PVDF) membranes (Millipore Corp). After incubation with 5% BSA in Tris Buffered Saline with Tween-20 (TBST) for 1 h, the membrane was incubated with the indicated antibodies overnight at 4 °C. The membrane was washed three times with TBST and then incubated with horseradish peroxidase-conjugated anti-rabbit antibodies (1:2000 dilution) for 1 h. The membrane was washed three times with TBST and visualized with the Bio-Rad system. The following primary antibodies were used: anti-C3aR, anti-p53, anti-p21, anti-p16, and anti-β-actin.

### Real-time PCR

Total RNA was extracted from cells *via* TRIzol reagent, and cDNA was reverse transcribed *via* HiScript III RT SuperMix (Vazyme) under the following conditions: 15 min at 37 °C and 5 s at 85 °C. 5 s cDNA was subsequently amplified *via* the use of ChamQ SYBR qPCR Master Mix (Vazyme). The transcript levels of each sample were normalized to those of β-actin and expressed as fold changes relative to the control levels shown.

### Statistical analysis

The data were analyzed with GraphPad Prism eight and are presented as the means ± SEMs. Student's *t* test was used when two conditions were compared. Multigroup comparisons of the means were carried out *via* one-way analysis of variance (ANOVA) with *post hoc* contrasts *via* the Student–Newman–Keuls test. Data analyses were repeated at least three times. The significance level was set at *p* < 0.05.

## Data availability

The data that support the findings of this study are available on request from the corresponding author.

## Supporting information

This article contains supporting information.

## Conflict of interest

The authors declare that they have no conflicts of interest with the contents of this article.

## Supporting information

Western blot
