# Supplementary material for: Knockdown of C3aR alleviates age-related bone loss via activation of YAP1/β-catenin signaling
Source: J Biol Chem. 2025 Apr 9;301(5):108500. doi: 10.1016/j.jbc.2025.108500 (PMC12139412; doi:10.1016/j.jbc.2025.108500)
Supplement: Western blot [file mmc1.docx]

**SUPPLEMENTARY TABLES**

Supplementary Table 1. Genes and primer sequences for qRT-PCR

| Gene |  | Primer sequence |
| --- | --- | --- |
| *Alp* | Forward | CCAACTCTTTTGTGCCAGAGA |
|  | Reverse | GGCTACATTGGTGTTGAGCTTTT |
| *Runx2* | Forward | AACGATCTGAGATTTGTGGGC |
|  | Reverse | CCTGCGTGGGATTTCTTGGTT |
